# *Allium fistulosum* L. Alleviates Apple Replant Disease by Suppressing *Fusarium solani*

**DOI:** 10.3390/jof8101071

**Published:** 2022-10-12

**Authors:** Weixiao Tang, Gongshuai Wang, Ran Chen, Xin Liu, Xuesen Chen, Xiang Shen, Chengmiao Yin, Zhiquan Mao

**Affiliations:** 1 College of Horticulture Science and Engineering, Shandong Agricultural University, Tai’an 271018, China; 2 State Key Laboratory of Crop Biology, College of Horticultural Science and Engineering, Shandong Agricultural University, Tai’an 271018, China; 3 College of Agricultural Science and Technology, Shandong Agriculture and Engineering University, Jinan 251100, China; 4 State Key Laboratory of Crop Biology, College of Agronomy, Shandong Agricultural University, Tai’an 271018, China

**Keywords:** ARD, *Fusarium solani* isolate HBH 08, GC–MS, dimethyl disulphide, diallyl disulphide, root exudates, qPCR

## Abstract

*Fusarium solani* has often been isolated from replanted apple roots, suggesting that it is associated with apple replant disease. The mechanism underlying the ability of the mixed cropping of apple trees with *Allium fistulosum* L. to alleviate apple replant disease remains unclear. The aim of this study was to determine the pathogenicity of the *Fusarium solani* isolate HBH 08 isolated from diseased roots and the effect of *A. fistulosum* L. and its root secretions on *Fusarium solani* isolate HBH 08 and apple seedings. The field experiment showed that *A. fistulosum* L. not only significantly reduced the amount of the *Fusarium solani* isolate HBH 08 in replanted soil but also increased the biomass of the grafted apple seedlings. The GC–MS analysis indicated that dimethyl disulphide and diallyl disulphide were active molecules in the root exudates of *A. fistulosum* L. They inhibited the growth of the *Fusarium solani* isolate HBH 08 mycelium and decreased the number of spores germinated. In addition, these compounds reduced the amount of the *Fusarium solani* isolate HBH 08 under replanted conditions and promoted the growth of grafted apple seedlings. Overall, mixed cropping with *A. fistulosum* L. might be an effective approach for cultivating apple trees and controlling apple replant disease.

## 1. Introduction

The poor growth of apple trees that occurs after replanting on a site where the same or closely related species were previously grown is referred to as “apple replant disease” or “replant disorder” [1]. The symptoms of apple replant disease (ARD) include necrosis of the fine feeder roots, stunted tree growth both above and below the ground, and drought and nutrient stress, leading to reduce yield. The effects of ARD on fruit yield can persist over the entire lifetime of the orchard [2], leading to substantial economic losses [3]. ARD is widespread and has been documented in all the major fruit-growing regions of the world, including the USA [4,5], Canada [6], Australia [7], Italy [8], South Africa [3], and China [9].

There are many factors that cause ARD, and most of the evidence indicates that the disease is primarily caused by biotic factors [1]. Numerous biological agents have been implicated as causes of ARD, including pathogenic nematodes [3,10], fungi [2,6,8,11] and bacteria [12]. The recent studies have suggested that pathogenic fungi such as *Phytophthora*, *Pythium*, *Cylindrocarpon*, and *Rhizoctonia* are associated with ARD [13,14]. The pathogenic fungi that cause ARD in different regions are different. The previous studies proved that *Fusarium* is one of the main pathogens causing ARD in China [15,16,17]. *Fusarium* produces mycotoxins that grow and multiply rapidly, posing health risks to both humans and animals [18,19,20]. *Fusarium* can infect specific parts of many plants causing various plant diseases [21]. Approximately half of the 40 species in the genus parasitize plants and cause root rots, vascular wilts, and storage rots [11,22]. The *Fusarium solani* species complex (FSSC) can also infect different host plants, causing necrosis of the plant tissues as well as varying degrees of wilting [23].

Fumigation is currently the most effective method for preventing and controlling ARD in replanted orchards [24]. However, increases in the cost of fumigation and decreases in the future availability of currently used fumigants might impede the continued use of this control method. In addition, the recent studies have demonstrated that the effects of fumigation in promoting plant growth and decreasing pathogen pressure in the treated soils are not permanent [25]. Consequently, sustainable and environmentally friendly control methods have received increased research attention. Biochar application has been shown to reduce the abundance of the pathogenic fungus *Fusarium* *solani* and promote the growth of apple plants [26,27]. Bioactive plant products, such as Brassicaceae [28], *Azadirachta indica* [29], *Tagetes erecta* [30], and mint [31] introduced as cover crops or soil amendments, have received increased interest for their ability to control the spread of soil-borne diseases. The incorporation of *Brassica napus* seed meal amendment into orchard soils alters soil microbial communities and reduces infections by pathogens in replanted soil [32]. Similar results have been obtained when *Tagetes erecta* is introduced as a cover crop before growing taro (*Colocastia esculenta*) [33]. However, the suppression of pathogens is limited by the concentration and perdurability of the bioactive plant products [34].

*Fusarium solani* has often been isolated from replanted apple roots in major fruit-growing regions in China, suggesting that it is associated with apple replant disease (ARD) [16,17]. An *Allium fistulosum*–*Brassica juncea*–*Triticum aestivum* rotation has been shown to promote the growth and yield of apple seedlings, reduce the abundance of harmful *F. solani* in soil, and suppress the growth of pathogenic fungi [35]. Mixed cropping of apple trees with *Allium fistulosum* L. has been shown to markedly alleviate ARD, but the underlying mechanism of this effect remains unclear. The *Allium* species contain a complex set of sulfur compounds with antimicrobial properties [36]. *A. fistulosum* L. has been shown to control bacterial wilt caused by *Ralstonia solanacearum* [37]. Onion and garlic crop residues have also been shown to reduce the germination of seeds of weedy annual plants [38]. Mixed cropping of *A. fistulosum* L. and apple seedlings has been shown to be an effective approach for improving the soil microbial environment and enhancing the growth of *Malus hupenhensis* Rehd. seedlings. However, the active molecules of *A. fistulosum* L. that inhibit the causal pathogens of ARD remain unknown. The aim of the study was to: (1) determine the pathogenicity of the *Fusarium solani* isolate HBH 08; (2) identify the active molecules released from the roots of *A. fistulosum* L.; and (3) evaluate the effect of active molecules on the *Fusarium solani* isolate HBH 08. We conducted the following experiments based on the hypothesis that “apple trees mixed with *Allium fistulosum* L. can alleviate apple replant disease”.

## 2. Materials and Methods

### 2.1. Fungal Pathogen Isolation and Identification

**Figure 1:**
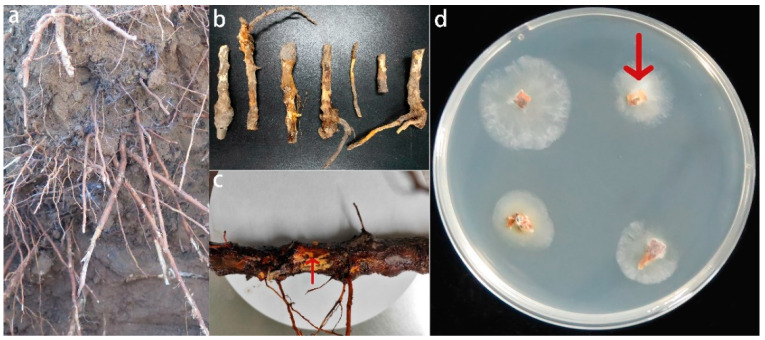
Diseased roots (**b**,**c**) collected from apple trees replanted from old orchards (**a**), the junction between diseased and healthy tissues on the bast of diseased roots has been marked with arrows (**c**), and fungal cultures grown from the junction between diseased and healthy tissues on PDA after 2–3 days at 28 °C, where the fungal culture indicated by the arrow is the subsequent isolation and identification of the *Fusarium solani* isolate HBH 08 (**d**).

Infected roots were collected from 4-year-old replanted apple trees in an old apple orchard that has been cultivating apples for more than 30 years in Tai’an, Shandong Province, China. The geographical coordinates of the sampling were 36°12′21.096″ N, 117°5′40.164″ E (Figure 1a). We selected one tree at the center of each of the four sides of the orchard and one tree at each of the four corners; one tree was selected in the main center of the orchard, and a total of nine trees were sampled. A five-point sampling method was used to sample five points within a 1 m diameter area centered on the trunk of each tree, and roots were collected at a depth of 10–40 cm.

The isolation procedure was based on that described in Wang et al. with slight modification of the separation scheme [39]. First, the roots were washed with deionized water, soaked in 75% alcohol for 10 min, soaked in 4% sodium hypochlorite for 10 min, soaked in sterile water for 10 min, and rinsed in sterile water 4–5 times. Next, the epidermis of the roots was removed using a sterilized scalpel, and the junction of diseased and healthy tissue in the bast (0.5–1 cm sections) was cut with a sterilized scalpel (Figure 1b) and placed on fresh potato dextrose agar (PDA) medium (4 mL of penicillin–streptomycin mixture per 1 L of medium) in Petri dishes (9 cm diameter) with four diseased root sections in each medium for 2–3 days in an incubator at 28 °C (Figure 1c). After 2–3 days, the mycelium grown from the diseased roots was removed with a sterilized toothpick and transferred to fresh PDA medium for purification using the three-zone delineation method until a single colony could be observed. An agar plug was then removed from a single colony with a sterilized toothpick and transferred to fresh PDA medium for pure culture.

Morphological identification of the isolated *Fusarium solani* isolate HBH 08 was carried out after incubation in an incubator at 28 °C for 5 days. The sequence numbers of the three identified sequences (ITS, TEF1α, RPB2) of *Fusarium solani* isolate HBH 08 on NCBI were OP268218, OP373202, and OP373203 (Table S2). For the identification of *Fusarium solani*, refer to the *Fusarium* laboratory manual [40]. Colony morphology, microconidia morphology, macroconidia morphology, chlamydospore morphology, and monophialides morphology were preliminarily characterized using BX-51 light microscopy.

Fungal DNA was extracted from pure 7-day-old mycelia using the Fungal DNA Kit (Omega Bio-Tek, Norcross, GA, USA) per the manufacturer instructions. The fungal internal transcribed spacer (ITS) region was amplified using the primers ITS1 and ITS4. According to the method of Arif et al., the primers TEF-Fs4f and TEF-Fs4r were used to amplify the fungal translation elongation factor 1-alpha (TEF1α) gene [41]. According to the method of O’Donnell et al., the primers RPB2-5F2 and RPB2-7cR were used to amplify the fungal RNA polymerase II second largest subunit (RPB2) gene [42]. The description of the DNA regions and primers used for molecular identification is shown in Table 1. The PCR amplification system contained 25 μL of 2 × PCR Master Mix, 1 μL of DNA, 1 μL of each primer (10 M), and 22 μL of ddH_2_O. PCR amplification was performed in a 2720 Thermal Cycler (Applied Biosystems, Waltham, MA, USA) with 3 min of pre-denaturation at 95 °C, 35 cycles of denaturation at 95 °C for 30 s, 35 cycles of annealing at 55 °C for 30 s, 30 s of extension at 72 °C, and 10 min of renaturation at 72 °C. PCR products were confirmed and purified from a 2.0% agarose gel using the DNA Fragment Purification Kit (TaKaRa Biotech Co., Ltd., Dalian, China) per the manufacturer instructions.

After purification, the amplicons were sequenced at Shanghai Sangon Co., Ltd. (Shanghai, China). The two DNA sequences of the isolates were sequenced against the National Center for Biotechnology Information (NCBI) database (http://www.ncbi.nlm.nih.gov, accessed on 31 July 2022) using Basic Local Alignment Search Tool (https://blast.ncbi.nlm.nih.gov/Blast.cgi, accessed on 1 August 2022) for sequence similarity searches.

### 2.2. Pathogenicity Testing

Liquid potato dextrose (PDB) medium was inoculated with *Fusarium solani* isolate HBH 08 and incubated at 28 °C in the dark for 4 to 7 days to prepare spores of the isolated and purified pathogenic fungus *Fusarium solani* isolate HBH 08, and the conidial suspension was filtered through three layers of gauze to separate the conidia from the mycelium. The concentration of the spore solution was adjusted to the desired concentration (10^6^ spores/mL) by counting spores in a hemocytometer with sterilized ultrapure water [43]. We selected 48 M9T337 seedlings (5–7 leaves) with similar growth status. A modified version of the dip-and-cut technique was used to inoculate M9T337 seedlings with *Fusarium solani* isolate HBH 08 by referring to the method of Sun et al. with minor modifications [44]. Before planting, the roots of M9T337 histoculture seedlings were washed and cut 1 cm with sterilized scissors. The roots of 24 histoponic seedlings were immersed in a conidial suspension (10^6^ spores/mL) for 10 min, and then the seedlings were transplanted into black plastic containers containing a sterilized soil and substrate mixture (1:1) and watered with 10 mL of 10^6^ spores/mL of the conidial suspension. The other 24 seedlings were immersed in sterile water for 10 min and transplanted and watered with the same volume of sterile water. Seedlings were incubated in a lighted incubator (25 °C, 12 h/12 h). After two weeks, seedlings watered with spore solution were defoliated and checked for leaf necrosis and root rot; the same fungal species used for inoculation experiments were reisolated from the roots as previously described to test the Koch hypothesis. The seedlings were also observed for wilting symptoms [45] and necrotic symptoms [46], and disease intensity was calculated based on the degree of wilting and necrotic symptoms [47].

### 2.3. Field Experiments

The field experiment was conducted in 2015 and 2016 in an old apple orchard that has been used for apple cultivation for more than 30 years in Tai’an, Shandong Province, China (36°12′21.096″ N, 117°5′40.164″ E). Soil samples were sandy with the following chemical characteristics: 7.8 mg∙kg^−1^ NO_3_^−^; 5.1 mg∙kg^−1^ NH_4_^+^; 26.7 mg∙kg^−1^ available P; 28.39 mg∙kg^−1^ available K; 23.1 mg∙kg^−1^ organic matter; and pH 5.9. Two-year-old Fuji apple trees on the rootstock *M. hupenhensis* Rehd. were planted in a site that previously had apple trees. The experiment was split into three treatments: (1) MB, replanted soil exposed to methyl bromide fumigation (high standard control); (2) MC, replanted soil subjected to mixed planting with *Allium fistulosum* L. (5 g of seeds were sown on each tree tray); and (3) UN, untreated replanted soil (control). Each treatment had 20 replications (20 young trees). In our experiment, the methyl bromide treatment served as a high standard control for measuring the degree to which mixed cropping improved replanted soil. 

A plot (40 m × 30 m) was divided into three equally sized areas designated as A, B, and C, which corresponded to the treatments MB, MC, and UN. In early March 2015, a subplot with in area A (40 m × 10 m) was exposed to fumigation treatment with methyl bromide at normal doses (50 g·cm^−2^ = 1 kg of methyl bromide per 20 m^2^), followed by mulching for one week and drying for one week; the soil was turned over three times during the drying period. Trenches were then dug at a spacing of 1.5 m × 4.0 m in areas A, B, and C, and two-year-old grafted young trees were planted in the trenches. In mid-May, 5 g of *A. fistulosum* L. seeds were sown within 40 cm of the area B tree trays. *A. fistulosum* L. plants were harvested in March of the following spring, and the seeds continued to be sown during this period. Samples were collected in September 2015 and 2016, and three young apple trees were randomly selected to serve as three replicates for each treatment. Plant height and fresh weight were measured with a meter stick and electronic scale, respectively. After removing topsoil, soil was collected at a depth of 5–20 cm and at a distance of 30 cm from the young trees; soil samples were taken at five different spots and pooled. The trees were carefully excavated; the roots were then rinsed with a hose and brought to the laboratory.

### 2.4. Design of Specific Primers and qPCR Specificity Analysis

Blast homology matching was performed in NCBI GenBank based on the ITS sequence of *Fusarium solani* isolate HBH 08; the specific primers FF (5′-CAAGCAAATAAATTAAAACTTTCA-3′) and FR (5′-CGTTCAAAGATTCGATGA-3′) were designed using Primer Premier 5.0 software and synthesized from Shanghai Sangon Co., Ltd. (Shanghai, China). The amplified target fragment was 122 bp in length. The purified DNA of *Fusarium solani* isolate HBH 08 was used as template for PCR amplification with specific primers. The PCR products were purified according to the instructions of the PCR product purification kit (TransGen Biotech Co., Ltd., Beijing, China), and then the purified PCR products were ligated to the PMD18–T vector (TaKaRa Biotech Co., Ltd., Dalian, China) according to the instructions. The ligation reaction system consisted of 1 μL of PMD18–T vector, 4 μL of purified PCR product, and 5 μL of Solution I. The ligation was performed at 4 °C for 4 h. The ligation products were transferred into DH5α *E. coli* receptor cells (TaKaRa Biotech Co., Ltd., Dalian, China). Positive clones were obtained after sequencing verification, cultured in LB liquid medium, and plasmids were extracted according to the method of the plasmid extraction kit. The plasmid concentration of the positive clone of *Fusarium solani* isolate HBH 08 was measured to be 19.7 ng/μL. The concentrations of the extracted plasmids were measured with an ultra–micro UV spectrophotometer ThermoNanoDrop 2000 and then diluted with sterile water to a 10^0^–10^−5^ ng/μL gradient of template, respectively. Real–time fluorescent quantitative PCR amplification was performed using a Bio–RadCFX96 quantitative PCR instrument with a two–step method. qPCR was performed with a 25 μL reaction system consisting of 12.5 μL SYBR green PCR Master Mix (TaKaRa Biotech Co., Ltd., Dalian, China) and 1 µL of upstream and downstream specific primers (10 µmol/L). The qPCR reaction conditions were: pre-denaturation at 95 °C for 30 s; denaturation at 94 °C for 5 s, annealing at 60 °C for 30 s, extension at 72 °C for 30 s, 40 cycles; and dissociation at 95 °C for 30 s, 60 °C for 15 s, linear increase from 60 °C to 95 °C within 10 min, and 95 °C for 15 s. Thus, Ct values and dissolution curves were obtained. The correlation coefficient and linear equation of the real-time PCR standard curve for *Fusarium solani* isolate HBH 08 were: R^2^ = 0.994, y= −2.352x + 26.941 where the logarithm of the plate copy number is the horizontal coordinate and the cycle number Ct value is the vertical coordinate (Figure S1A). Further analysis of the melting curve (Figure S1B) showed that the melting curve of the amplified product had a single peak pattern and did not produce a peak pattern caused by primer dimer, indicating that this primer was specific and had no nonspecific amplification products.

### 2.5. Collection, Identification, and Quantification of the Root Exudates in A. fistulosum L. by GC-MS

Two-month-old *A. fistulosum* L. plants were used to collect root exudates by hydroponics. Twenty plants were planted in a plastic box with 1 L of deionized water for 48 h to simulate soil conditions. Crude roots were extracted by ethyl acetate (1:1 *v/v*) three times. The solution was further concentrated under reduced pressure at 30 °C until it was nearly dry, then 5 mL of ethyl acetate was added to dissolve the sample, and the solution was passed through a 0.45-µm organic membrane filter. The sample was then subjected to GC–MS analysis. 

The analysis of all essential root exudates was performed using a GC/MS-QP2010Plus, equipped with an Rtx-5MS capillary column (30 m, 0.32 mm i.d., 0.25 µm film thickness). The carrier gas was helium, and the flow rate was 2.4 mL/min. The column temperature was initially 50 °C for 2 min, and then it was gradually increased to 250 °C at 6 °C/ min, and the duration of the temperature program was 10 min. The injection port temperature was 230 °C. The mass spectrometer was operated in the electron impact ion (EI) mode with a source temperature of 200 °C. The electron energy was 70 eV. The MSD was run in full scan mode over 45–550 amu. The compounds were identified by their mass spectra, retention time, and standard references through GC–MS analysis. Pure reagent dimethyl disulphide (DMDS) and diallyl disulphide (DADS) (Sigma, St. Louis, MI, USA) were used as standard substances to quantify their concentrations. 

### 2.6. Effects of the Root Exudates on the Mycelial Growth and Spore Germination of Fusarium solani Isolate HBH 08

Based on our preliminary study, DMDS and DADS were quantified in the root exudates of *A. fistulosum* L. by GC-MS. The experiment was divided into six treatments, with three replicates for each treatment. Treatments included: RE, *A. fistulosum* L. root exudates by hydroponics; LCDMDS (low concentration of dimethyl disulphide), measured concentrations of DMDS (22.4 μg/mL); HCDMDS (highly concentrated dimethyl disulphide), 5 times the measured concentration of DMDS (112 μg/mL); LCDADS (low concentration of diallyl disulphide), measured concentrations of DADS (15.2 μg/mL); HCDADS (highly concentrated diallyl disulphide), 5 times the measured concentration of DADS (76 μg/mL); and UN, untreated replanted soil (control). DMDS and DADS were applied to potato dextrose agar (PDA) medium to prepare medium containing different concentrations of DMDS, DADS, and root exudates. 

The cakes of *Fusarium solani* isolate HBH 08 were placed on the prepared PDA medium. They were then incubated at 28 °C for 5 to 7 days. Sterile water was used as a control. The growth diameter of *Fusarium solani* isolate HBH 08 was measured when the control growth diameter covered two-thirds of the culture dish. The cross-crossing method was used to measure the colony growth diameter and calculate the inhibition rate by referring to the method of Xie et al. [48]. 

*Fusarium solani* isolate HBH 08 conidia suspension (10^6^ spores/mL) was prepared to determine the effects of sulfur compounds and root exudates on spore germination. Conidial suspensions of *Fusarium solani* isolate HBH 08 were treated with sulfur compounds and root exudates of *A. fistulosum* L. and then incubated at 25 °C for 7 h. After 7 h, 0.1% HgCl_2_ was added to all the experimental groups to stop spore germination, and the spore germination rate was calculated. Each treatment had three replicates.

### 2.7. Quantitative Real-Time PCR (qPCR) of Fusarium solani Isolate HBH 08 in Replanted Soil Treaded with Root Exudates

In the pot experiment, there were 10 replicates of pots per treatment (diameter 27 cm, height 25 cm) filled with approximately 6 kg of soil (a total of 50 pots) at Shandong Agricultural University (Tai’an, Shandong Province, China). The soil samples were collected from a 30-year-old apple orchard in Tai’an at a depth of 5–50 cm (36°12′21.096″ N, 117°5′40.164″ E). *M. hupenhensis* Rehd. seedlings with four leaves were planted in each pot. Treatments contained: (1) replanted soil treated with 200 mL of DMDS (22.4 μg/mL); (2) replanted soil treated with 200 mL of DADS (15.2 μg/mL); (3) replanted soil treated with 200 mL of root exudates of *A. fistulosum* L.; (4) replanted soil fumigated with methyl bromide; and (5) untreated replanted soil. Samples were taken after 5 months of seedling growth and measured in the same way as in Section 2.3. Fungal DNA was obtained from rhizospheric soil using the Power Soil DNA Isolation Kit (MoBio, San Mateo, CA, USA), and soil DNA was extracted per the manufacturer instructions.

### 2.8. Statistical Analysis

Growth data were presented as the mean ± standard error of three replicates. Data were collated using Microsoft Excel 2003 software (version 2003, Microsoft Inc., Redmond, WA, USA). Plots for data analysis were produced using GraphPad Prism8 software (version 8.0, GraphPad Software Inc., San Diego, CA, USA). Statistical analyses, such as analysis of variance (ANOVA), were performed using SPSS software (version 19.0, SPSS Inc., Chicago, IL, USA). The phylogenetic tree was constructed by MEGA 7.0 software (version 7.0, Mega Limited, Auckland, New Zealand). Duncan’s multiple range test was used to compare significant differences between treatments.

## 3. Results

### 3.1. Isolation, Morphological Identification, and Pathogenicity Assays of the Fusarium solani Isolate HBH 08

We isolated and identified fungi from an orchard. Twenty-one genera of 149 fungi were isolated from 135 roots of nine trees, and the percentage of each genus and the percentage of the different *Fusarium* species in the genus *Fusarium* are shown in Figure S2. Among them, three species of *Fusarium solani* were isolated.

**Figure 2:**
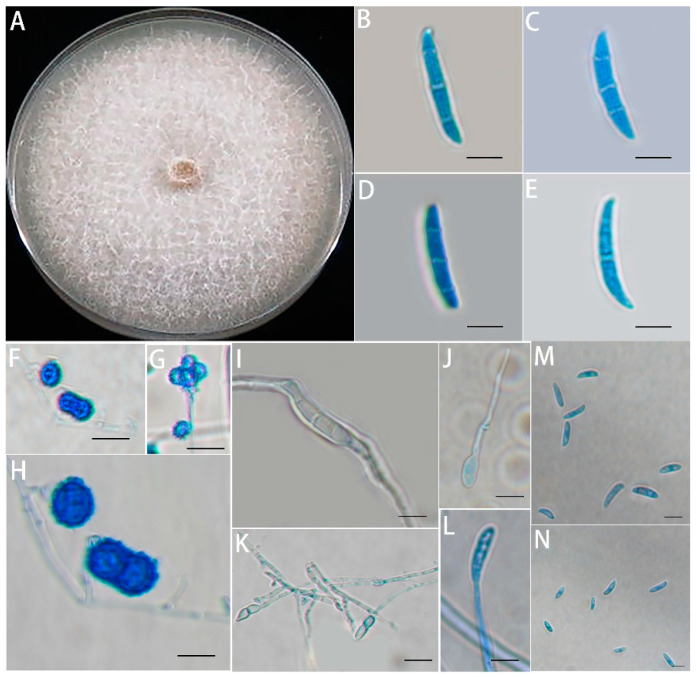
Culture characteristics and microscopic appearance of isolates. (**A**) Appearance of isolates in pure culture on potato dextrose agar medium; (**B**–**E**) macroconidia; (**F**–**H**) chlamydospores; (**I**–**L**) monophialides; and (**M**,**N**): microconidia. Scale bars: (**F**,**G**,**K**) 20 μm; (**B**–**E**,**H**–**J**,**L**–**N**) 10 μm.

Next, we examined the morphology of an isolate purified from the diseased roots in the orchard. One of the strains grew rapidly on PDA, reaching a colony diameter of 85 mm after 7 days of growth at 28°C. The colonies were white to cream in color with sparse mycelium on them (Figure 2A). The macroconidia were relatively long and stout with multiple septa, bluntly rounded apical cells, and poorly developed basal cells (Figure 2B). The chlamydospores were usually single or in pairs or clusters, produced in the middle of the mycelium or at the ends of short lateral branches and were globular and ellipsoidal, with rough walls (Figure 2C). The monophialides were single spores, and the length of their germination period was long (Figure 2D). The microconidia were numerous and oval, reniform, ovate, and fusiform in shape, with no septa or one septum (Figure 2E). The pathogen isolated from the infected roots was preliminarily identified as *Fusarium solani* based on its morphology.

**Figure 3:**
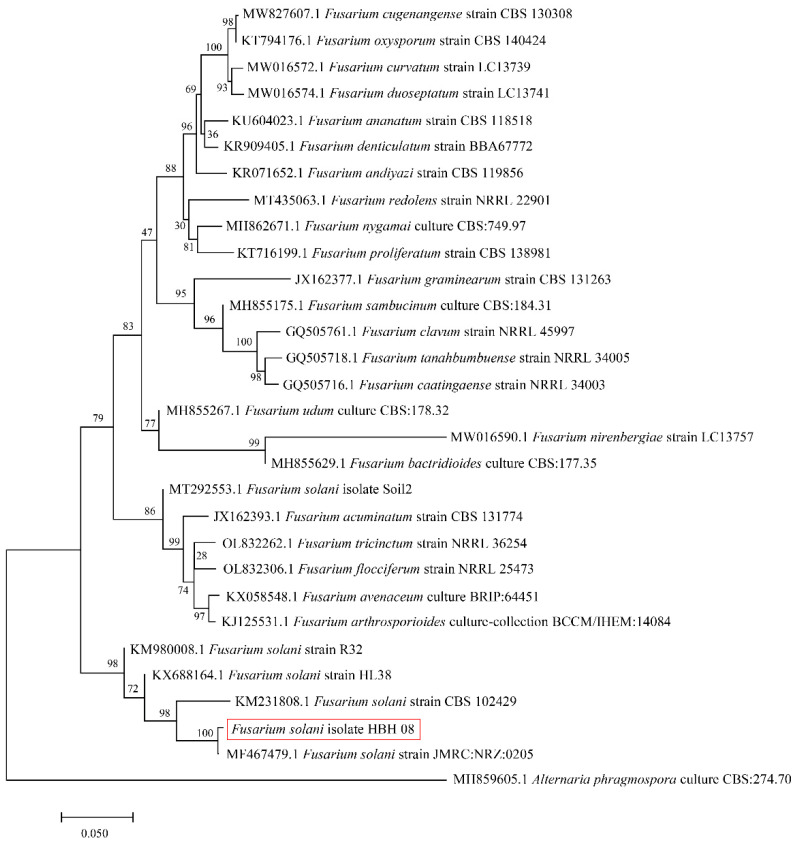
Phylogenetic tree constructed from the ITS sequence, TEF-1α sequence, and RPB2 sequence of the isolated and identified fungal pathogen *Fusarium solani* isolate HBH 08. Data for other strains of different species were obtained from the NCBI GenBank. The developmental tree is drawn to scale, with branch lengths at the same evolutionary distances as the phylogenetic tree, and the marker lengths in the lower left corner are evolutionary distances.

The phylogenetic tree (Figure 3) was constructed by the neighbor-joining method in MEGA 7.0 software using the data obtained from the NCBI GenBank using BLAST. The results established from the ITS sequence, TEF-1α sequence, and RPB2 sequence splicing of this strain showed that this strain was most closely related to *Fusarium solani*. Therefore, the strain was identified as *Fusarium solani* based on the characterization and clustering similarity.

**Figure 4:**
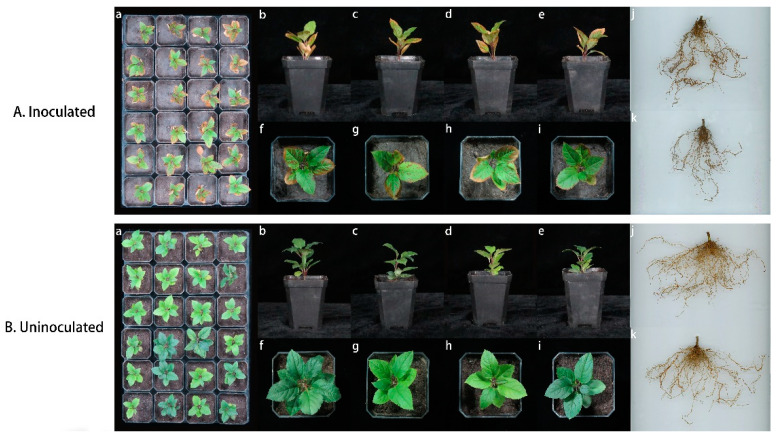
Effect of M9T337 inoculated with the *Fusarium solani* isolate HBH 08 (**A**) and without the *Fusarium solani* isolate HBH 08 (**B**). A: aboveground (**a**–**i**) and root system (**j**,**k**) incidence of M9T337 inoculated with the *Fusarium solani* isolate HBH 08, (**B**): aboveground (**a**–**i**) and root system (**j**,**k**) growth control of M9T337 without the *Fusarium solani* isolate HBH 08.

In addition, the results of the pathogenicity assays revealed that the seedlings of M9T337 gradually stopped growing after irrigation of the planting substrate with a conidial suspension compared with the control. After 2 weeks, the edge of the basal blade appeared yellow, the disease intensity for the degree of seedling wilting was 70.8%, and the disease intensity for the degree of seedling necrotic was 67.5%. In addition, root rot was evident in some seedlings (Figure 4), and the same fungal species used for the inoculation experiments were re-isolated from the roots that had decayed, thus conforming to the Koch hypothesis. Therefore, the *Fusarium solani* isolate HBH 08 was pathogenic to the apple seedlings.

### 3.2. Effect of Mixed Cropping of Grafted Apple Seedlings with A. fistulosum L. on the Biomass of Grafted Apple seedlings and Fusarium solani Isolate HBH 08 

The plant height and fresh weight of the grafted seedlings were measured in September 2015 and 2016 (Table 2). The ANOVAs showed that the height and fresh weight of the grafted seedlings were highest for soil fumigated by methyl bromide (MB) (*p* < 0.005), followed by grafted seedlings mixed-cropped with *A. fistulosum* L. (MC) in the field experiment. Compared with the grafted seedlings in untreated replanted soil (UN), the height and fresh weight of the grafted seedlings mixed-cropped with *A. fistulosum* L. (MC) were significantly increased by 17.06% and 70.19% in 2015 and by 18.34% and 82.93% in 2016, respectively. 

**Figure 5:**
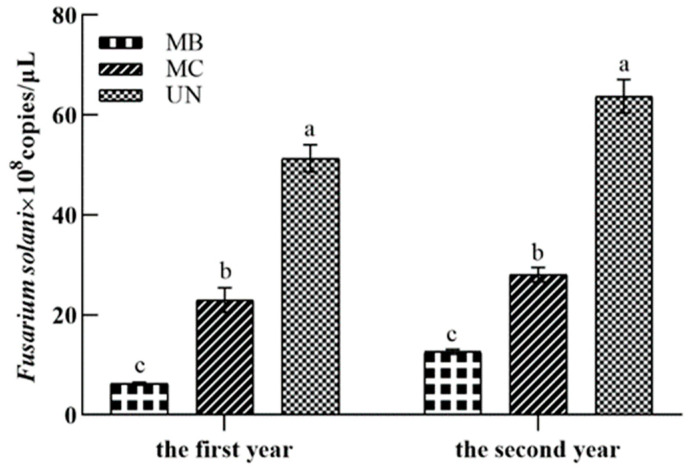
Effect of mixed cropping grafted apple seedlings for two years with *A. fistulosum* L. on the *Fusarium solani* isolate HBH 08 in the soil. MB, replanted soil treated with methyl bromide fumigation (high standard control); MC, replanted soil with *Allium fistulosum* L. (5 g of seeds were sown on each tree tray.); and UN, replanted soil as a control. Different letters (a, b, and c) indicate significant differences according to Tukey’s HSD test.

qPCR was used to determine the biomass represented by the fungal DNA of the *Fusarium solani* isolate HBH 08 in the replanted soil. The real-time fluorescence units were plotted against the initial concentration of plasmid DNA, which ranged from 26.3 ng/μL to 2.63 × 10^−5^ ng/μL. The standard curve produced by the real-time PCR assay revealed strong linearity within the detection limit and a high correlation between the Ct values (R^2^ > 0.99). The standard curve was y = −2.362x + 27.479 (R^2^ = 0.994). qPCR analysis revealed a significant reduction of 55.17% in the first year (*p* < 0.05) and 55.98% in the second year (*p* < 0.05) in the amount of the pathogenic *Fusarium solani* isolate HBH 08 in soil planted with the grafted seedlings mixed with *A. fistulosum* L. compared with the untreated replanted soil (Figure 5).

### 3.3. Quantification of Root Exudates and Their Effects on the Fusarium solani Isolate HBH 08

A total of 57 elements were identified in the root exudates of *A. fistulosum* L. by the GC–MS analysis, including hydrocarbons, esters, benzene, alcohol, naphthalene, phenol, thioether, heterocyclic, and other compounds (Table 3). N’-methyl-N and N-diphenyl- were dominant in the root exudates of *A. fistulosum* L. Benzene compounds were more abundant in the root exudates, such as m-xylene (8.02 %), o-xylene (3.93%), and ethylbenzene (3.25%). Hydrocarbons and esters were also abundant in the root exudates of *A. fistulosum* L.

DMDS and DADS were detected in the root exudates of *A. fistulosum* L. The quantitative analysis with GC–MS revealed that the content of DMDS was 22.4 μg/mL, and the content of DADS was 15.2 μg/mL.

**Figure 6:**
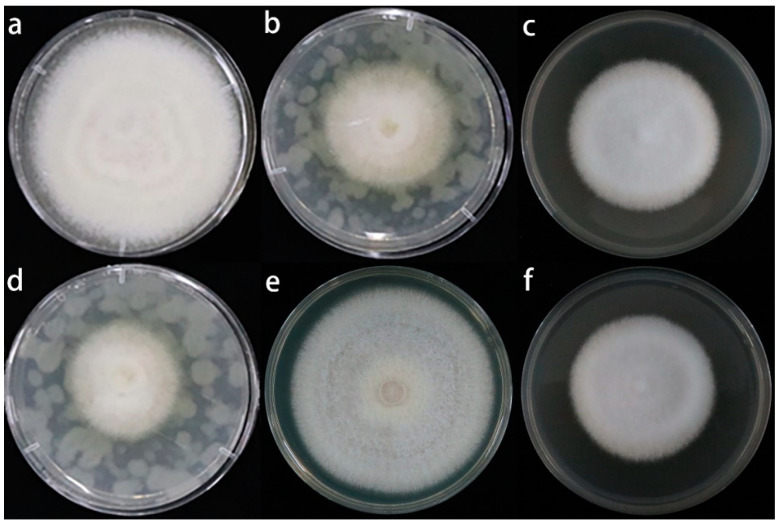
Effect of sulfur compounds and root exudates of *A. fistulosum* L. on the growth of the *Fusarium solani* isolate HBH 08. (**a**) UN, untreated replanted soil (control); (**b**) RE, *A. fistulosum* L. root exudates by hydroponics; (**c**) LCDMDS, measured concentrations of DMDS (22.4 μg/mL); (**d**) HCDMDS, 5 times the measured concentration of DMDS (112 μg/mL); (**e**) LCDADS, measured concentrations of DADS (15.2 μg/mL); (**f**) HCDADS, 5 times the measured concentration of DADS (76 μg/mL).

**Figure 7:**
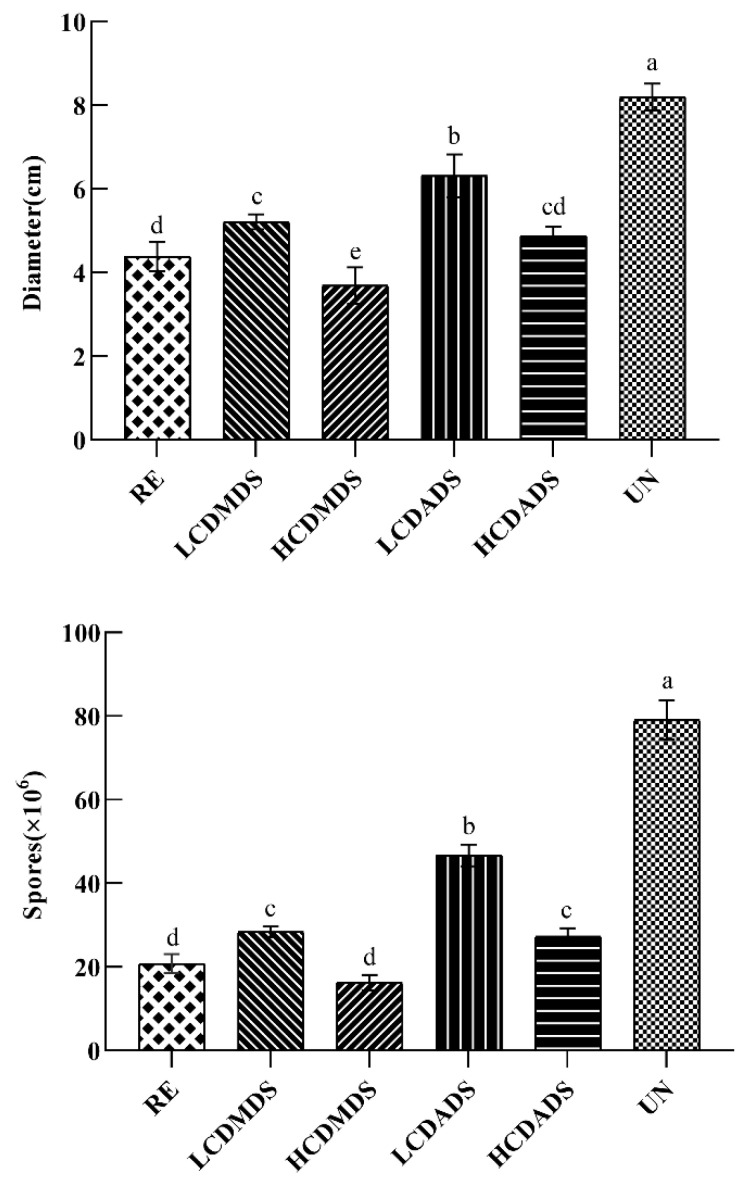
The effect of the sulfur compounds and root exudates of *A. fistulosum* L. on the growth of the *Fusarium solani* isolate HBH 08 and on the spore germination of the *Fusarium solani* isolate HBH 08. RE, *A. fistulosum* L. root exudates by hydroponics; LCDMDS, measured concentrations of DMDS (22.4 μg/mL); HCDMDS, 5 times the measured concentration of DMDS (112 μg/mL); LCDADS, measured concentrations of DADS (15.2 μg/mL); HCDADS, 5 times the measured concentration of DADS (76 μg/mL); and UN, untreated replanted soil (control). Different letters (a, b, c, d, and e) indicate statistically significant differences according to Tukey’s HSD test (*p* ≤ 0.05).

Treatment with the single pure sulfur compounds and root exudates of *A. fistulosum* L. inhibited the growth of the *Fusarium solani* isolate HBH 08 mycelia (Figure 6 and Figure 7). The root exudates of *A. fistulosum* L. inhibited the mycelial growth by 46.48%, and the single sulfur compounds (LCDMDS, HCDMDS, LCDADS, and HCDADS) inhibited the mycelial growth by 36.36%, 54.94%, 20.94%, and 40.55% compared with the untreated replanted soil, respectively. The DMDS, DADS, and root exudates of *A. fistulosum* L. had a significant inhibitory effect on the spore germination capacity of the *Fusarium solani* isolate HBH 08 (Figure 7). The inhibition of the spore germination ability of the *Fusarium solani* isolate HBH 08 by the root exudates of *A. fistulosum* L., LCDMDS, HCDMDS, LCDADS, and HCDADS was 73.7%, 63.99%, 79.43%, 40.94%, and 65.38% compared with the untreated replanted soil, respectively. 

### 3.4. Effect of the Sulfur Compounds and Root Exudates of A. fistulosum L. on the Growth of M. hupenhensis Rehd. Seedlings and the Fusarium solani Isolate HBH 08 in Replanted Soil

**Figure 8:**
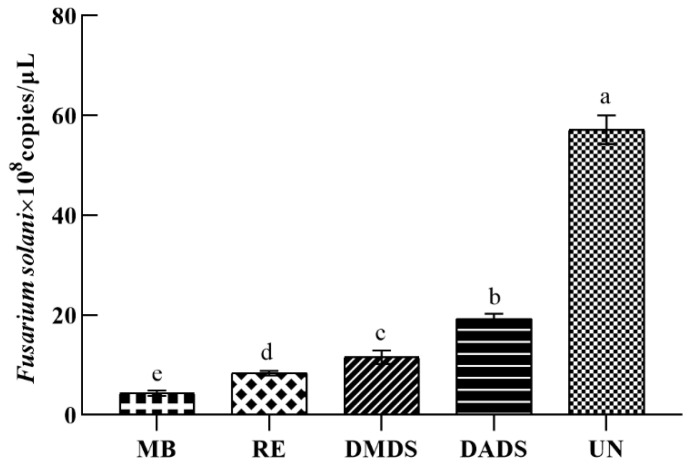
Effect of sulfur compounds and the root exudates of A. fistulosum L. on the abundance of the *Fusarium solani* isolate HBH 08. MB, replanted soil fumigated with methyl bromide; DMDS, replanted soil treated with dimethyl disulphide; DADS, replanted soil treated with diallyl disulphide; RE, replanted soil treated with root exudates; and UN, untreated replanted soil (control). Different letters (a, b, c, and d) indicate statistically significant differences according to Tukey’s HSD test (*p* ≤ 0.05).

After treatment with sulfur compounds and root exudates for 5 months, the plant height and fresh weight were measured. The results of the pot experiment indicated that the root exudates and single sulfur compounds (DMDS and DADS) had a significant effect on the growth of *A. fistulosum* L. compared with the untreated soil (Table 4). The treatment of the seedlings with the root exudates, DMDS, and DADS increased the seedling height by 65.42%, 35.51%, and 28.97% (P<0.005), respectively. The variation in the fresh weight among the treatments was similar to that observed for height. Furthermore, the root exudates and single chemicals (DMDS and DADS) also decreased the abundance of the *Fusarium solani* isolate HBH 08 compared with the untreated soil (Figure 8). The root exudates resulted in a reduction of 85.32% in the abundance of the *Fusarium solani* isolate HBH 08. The DMDS resulted in a reduction of 79.77% in the abundance of the *Fusarium solani* isolate HBH 08. The DADS resulted in a reduction of 66.25% in the abundance of the *Fusarium solani* isolate HBH 08.

## 4. Discussion

Methyl bromide is an effective fungicide. Crops planted in soil fumigated with methyl bromide showed significant improvements in growth. Similar results were obtained by van Schoor and Tewoldemedhin [3,49]. Several studies have found that *Pythium*, *Phytophthora*, *Rhizoctonia*, and *Cylindrocarpon* spp. are consistently isolated from replant soils, and pathogenicity tests suggest that these fungi might contribute to the development of ARD [13,14]. However, the causal factors of ARD vary from region to region [9,50]. Franke-Whittle et al. suggested that *Acremonium*, *Cylindrocarpon*, and *Fusarium* spp. were negatively correlated with growth height in replanted apples based on high-throughput pyrosequencing analysis [12]. The *Fusarium* species diversity has been suggested to be the cause of the crown rot of wheat in Serbia [51]. In India, *Fusarium sacchari* was identified as the causal micro-organism, and *Fusarium sacchari* and *Fusarium proliferatum* were found to be associated with *Pokkah boeng*, a Fusarium disease of sugarcane [52]. The Fusarium infestation of cells results in the secretion of a fungal toxin that binds to a protein in the cell protoplasmic membrane, which induces structural changes in the membrane, changes in membrane permeability, leakage of electrolytes, and damage to the cell, eventually resulting in plant disease and even death [53,54]. The *Fusarium solani* species complex (FSSC) has specific virulence factors residing on CD supernumerary chromosomes that are capable of causing disease in a variety of plants [55]. *F. solani*, which is an important soil-borne disease, can cause disease in several economically important crops, including the common bean [56], peanut [57], soybean [58], and sweet potato [59]. In addition, *Fusarium* spp. have been shown to induce the severe root death of apples in China [11,60]. In this study, the *Fusarium solani* isolate HBH 08 was isolated from the infected roots of replanted apple seedlings and tested for pathogenicity; our findings indicated that this pathogen causes leaf yellowing, severe root rot, and eventually the death of M9T337 seedlings. The qPCR results also indicated that the *Fusarium solani* isolate HBH 08 is abundantly present in untreated continuous cropping soil. The study indicated that the *Fusarium solani* isolate HBH 08 is an aggressive pathogen that can induce ARD.

Soil-borne diseases can generate a negative plant–soil feedback that leads to reduced crop yields and increases the prevalence of soil-borne diseases [61]. Controlling soil-borne fungal diseases is extremely difficult because pathogenic fungi have extensive mycelia and large numbers of spores. Environmental protection measures have been frequently used to prevent and control soil-borne plant diseases, and environmental protection measures have become environmentally friendly alternatives to synthetic pesticides. Composting [62,63], intercropping [64], and biofumigants [65] have been frequently used to control soil-borne plant diseases. Bioactive plant products have been increasingly used as cover crops and soil conditioners as biofumigants to mitigate ARD. Many studies have shown that *Allium* crops are biologically active plant materials that can promote crop growth and improve soil conditions [34,63]. The re-release of active hydrolysis products from bioactive plant products is short-lived due to their volatilization and degradation. For example, the effectiveness of allyl isothiocyanate, one of the active hydrolysis products released from soil amendments by the seed powder of *Brassica juncea*, lasts only 24–48 h [66]. The addition of *Allium ascalonicum* L. inhibits the enzymatic browning of apples, which increases antioxidant activity and enhances the nutritional quality of apples [67]. *A. fistulosum* L. mixed with apple seedlings reduces the number of harmful fungi and increases the number of bacteria, thus reducing ARD [68]. Hydroponic extracts of *A. fistulosum* inhibit the growth of *Ralstonia solanacearum*, and the treatment of the soil with these extracts significantly reduces the number of *R. solanacearum* [37]. *Allium fistulosum–Brassica juncea–Triticum* aestivum rotation in one year has been shown to increase the biomass of apple seedlings and reduce the number of harmful *Fusarium* spp. in soil, thus improving the community structure of soil micro-organisms [35]. In our two years of field trials, the biomass of apple grafted seedlings mixed with *A. fistulosum* L. increased, and the amount of *Fusarium solani* isolate HBH 08 in the soil decreased compared with the control, which alleviated the extent of soil ARD.

The protective effect of *Brassicaceae* residues against soil-inhabiting fungal plant pathogens and insect pests has been shown to last for weeks [69]. After biofumigation, pathogens can also recover and lead to root infections [70]. In addition, some studies have suggested that biofumigation substrates can sustain the saprophytic growth of plant pathogenic fungi and oomycetes and increase the pathogen inoculum potential [71,72,73,74]. However, living plants that continuously release volatiles belowground are effective agents for the biological control of soil-borne pathogens for long periods if the pathogens are susceptible to volatiles. In this study, root volatiles accumulated in the rhizosphere soils of the grafted apple seedlings mixed with *A. fistulosum* L., which inhibited the reproductive growth of fungal hyphae and conidia. Sulfur-containing salts might be as effective as synthetic fungicides for controlling the activity of pathogenic fungi [75]. Griseofulvin derivatives also show antifungal activity [76]. Members of the family *Allium*, including *A. fistulosum*, Chinese chive, and *A. cepa* contain complex biochemical compounds, such as sulfur compounds, with biocidal properties [36]. Most previous studies have focused on examining the use of *Allium* plants to control soil-borne plant pathogens [34]. The by-products of onions contain dimethyl disulphide and dipropyl disulphide, and the application of compost derived from onions to soil not only inhibits the growth and proliferation of *Pythium ultimum* but also increases asparagus and strawberry production [34]. In addition, 1-dimethyl-2-pentenal and dimethyl trisulfide from the leaves and roots of Chinese chive volatiles strongly inhibit the growth of *F. oxysporum* and can control Panama disease [64]. Whether the alleviation of ARD in the grafted seedlings mixed-cropped with *A. fistulosum* L. stems from the inhibition of the activity of pathogenic bacteria by the root exudates secreted by *A. fistulosum* L. remains unclear. However, the active constituents of the root exudates of *A. fistulosum* L. have not been identified. Whether these active ingredients show antimicrobial activity against *Fusarium solani* also remains unclear. Based on the GC–MS analyses of the pure compounds, two sulfur-containing volatiles from the roots of *A. fistulosum* L., including DMDS and DADS, were identified. Further analysis in subsequent experiments confirmed that the decrease in the abundance of the *Fusarium solani* isolate HBH 08 in the replanted soil mixed-cropped with *A. fistulosum* L. was related to the suppression of the sulfur-containing compounds in the root exudates. The experimental results showed that the growth of the *Fusarium solani* isolate HBH 08 and the germination of the microbial spores were dramatically inhibited by the single sulfur-containing compounds and the root exudates of *A. fistulosum* L. We found that the inhibitory effects of the single sulfur-containing compounds on the hyphal growth and germination of the *Fusarium solani* isolate HBH 08 increased as the concentration of these applied compounds increased, which was consistent with the results of Arnault [36]. However, the root exudates of *A. fistulosum* L. were more effective inhibitors of the growth of the *Fusarium solani* isolate HBH 08 compared with the single sulfur-containing compounds, which suggests that the root exudates consisted of other active substances that contribute additive, antagonistic, and synergistic effects. The qPCR results also revealed a significant reduction in the number of *Fusarium solani* isolate HBH 08 in replanted soils treated with root exudates and two single sulfur-containing compounds. Our study showed that root exudates and two sulfur-containing volatiles from the roots of *A. fistulosum* L., including DMDS and DADS, inhibited the activity of the *Fusarium solani* isolate HBH 08, which reduced the amount of the *Fusarium solani* isolate HBH 08 in the replanted soil, increased the biomass of plants, and slowed ARD. As the root exudates of *A. fistulosum* L and two sulfur-containing volatiles are broad-spectrum fungicidal substances that may inhibit a wide range of pathogens, it is also possible that ARD was alleviated because of its inhibitory effect on a combination of pathogens and not just on one strain of the *Fusarium solani* isolate HBH 08. More work will be continued in our future studies.

## 5. Conclusions

The *Fusarium solani* isolate HBH 08 has a strong pathogenic effect on *M. hupenhensis* Rehd. seedlings. The rapid diagnosis of the disease can be achieved through gene amplification, which is important for controlling the source of infection at the initial stage of the disease and reducing the epidemic harm of the disease. The results indicate that mixed cropping with *A. fistulosum* L. is a feasible and effective measure for circumventing the challenges posed by the *Fusarium solani* isolate HBH 08 for continuous apple cropping. However, additional studies are needed to evaluate the effect of mixed cropping with *A. fistulosum* L. on microflora and interactions with apple trees.

## 6. Patents

There are no patents resulting from the work reported in this manuscript.

## Figures and Tables

**Table 1:** Primers used for PCR and DNA sequencing.

Locus	Gene Product	Primer	Sequence (5′-3′)
ITS	Internal transcribed spacer	ITS1	TCCGTAGGTGAACCTGCGG
		ITS4	TCCTCCGCTTATTGATAGC
TEF1α	Translation elongation factor 1-alpha	TEF-Fs4f	ATCGGCCACGTCGACTCT
		TEF-Fs4r	GGCGTCTGTTGATTGTTAGC
RPB2	RNA polymerase II second largest subunit	RPB2-5F2	GGGGWGAYCAGAAGAAGGC
		RPB2-7cR	CCCATRGCTTGYTTRCCCAT

Degenerate sites in bold indicate the following: R = A or G; W = A or T; Y = C or T.

**Table 2:** Effect of mixed cropping with *A. fistulosum* L. on the biomass of the grafted apple seedlings.

Sampling Time	Treatment	Plant Height (cm)	Fresh Weight (kg)
The first year	MB	196 ± 3.46 a	5.10 ± 0.11 a
	MC	180.67 ± 4.91 b	4.07 ± 0.23 b
	UN	154.33 ± 2.97 c	2.39 ± 0.13 c
The second year	MB	256.67 ± 7.45 a	8.50 ± 0.32 a
	MC	247.33 ± 5.90 a	6.57 ± 0.34 b
	UN	209.00 ± 4.04 b	3.59 ± 0.27 c

MB, replanted soil treated with methyl bromide fumigation (high standard control); MC, replanted soil with *Allium fistulosum* L. (5 g of seeds were sown on each tree tray.); and UN, untreated replanted soil (control). Different letters (a, b, and c) indicate statistically significant differences according to Tukey’s HSD test (*p* ≤ 0.05).

**Table 3:** Chemical components in the root exudates of *A. fistulosum* L.

Compound	Relative Content %	Compound	Relative Content %
2,6,10-Trimethyldodecane	0.31	Hexadecanoic acid, methyl ester	0.51
2-Bromotetradecane	0.31	Dibutyl phthalate	0.46
3-Ethyl-3-methylheptane	0.11	Acetic acid, n-octadecyl ester	0.23
Cyclononasiloxane, octadecamethyl-	0.33	Sulfurous acid, diisobutyl ester	0.18
Decane	0.14	Benzene, 1,2,4,5-tetramethyl	0.34
Decyl iodide	0.27	Benzene, pentamethyl-	0.38
Dodecane, 4,6-dimethyl-	0.47	Ethylbenzene	3.25
Dodecane	0.46	M-xylene	8.02
Eicosane	0.32	O-Xylene	3.93
Heptadecane, 8-methyl-	0.21	Benzyl alcohol	0.64
Hexadecane	0.61	Isotridecanol-	0.36
Hexane, 3,3-dimethyl-	0.45	Methanethiol	1.84
Nonane, 5-methyl-5-propyl-	0.21	Dithioglycol	1.02
Octane, 2,3,6,7-tetramethyl-	0.26	Naphthalene	0.51
Pentadecane, 4-methyl-	0.19	Naphthalene, 1-methyl- a-	1.81
Tetradecane, 4-methyl-	0.33	Naphthalene, 2,3-dimethyl-	0.33
Tetradecane	0.71	Naphthalene, 2,6-dimethyl-	0.42
Undecane, 4,8-dimethyl-	0.28	Phenol, 2,2’-methylenebis [6-(1,1-dimethylethyl)-4-methyl-	0.2
Nonadecane, 4-methyl-	0.36	Phenol, 2,6-bis(1,1-dimethylethyl)-	0.16
Dotriacontane	0.74	Phenol, 3-pentadecyl- 3-	0.4
1,2-Benzenedicarboxylic acid, diisooctyl ester	0.77	Dimethyl disulphide	1.04
1-Hexadecanol, acetate	0.37	Diallyl disulphide	0.7
Diethyl phthalate	0.15	Dibenzofuran	0.29
Diisooctyl phthalate	2.26	Fluorene	0.39
Heptadecanoic acid, 16-methyl-, methyl ester	0.18	Urea, N’-methyl-N, N-diphenyl-	50.61
Methyl 13-methyltetradecanoate	0.57	2-Naphthalenamine, N-phenyl- N-	0.98
Octadecanoic acid, methyl ester	0.42	Methanesulfonylacetic	0.86
Phthalic acid, diisobutyl ester	1.36	Others	6.99

**Table 4:** Effect of root exudates *A. fistulosum* L. on the growth of *M. hupenhensis* Rehd. seedlings.

Treatment	Plant Height (cm)	Fresh Weight (g)
MB	74.67 ± 3.28 a	63.78 ± 4.08 a
DMDS	48.33 ± 1.86 c	36.75 ± 1.82 b,c
DADS	46.00 ± 1.73 c	31.29 ± 0.85 c
RE	59.00 ± 2.31 b	40.92 ± 3.82 b
UN	35.67 ± 1.76 d	21.88 ± 1.35 d

MB, replanted soil fumigated with methyl bromide; DMDS, replanted soil treated with dimethyl disulphide; DADS, replanted soil treated with diallyl disulphide; RE, replanted soil treated with root exudates; and UN, untreated replanted soil (control). Different letters (a, b, c, and d) indicate statistically significant differences according to Tukey’s HSD test (*p* ≤ 0.05).

## Data Availability

Data are contained within the article. All data and material are available.

## Supplementary Materials

The following supporting information can be downloaded at: https://www.mdpi.com/article/10.3390/jof8101071/s1, Figure S1: A: The standard curve for *Fusarium solani* isolate HBH 08 qRT-PCR; B: The melting curve for *Fusarium solani* isolate HBH 08 qPCR product; Figure S2: A: Relative abundance of 21 fungal genera isolated from diseased roots of apple trees; B: Percentage of isolation of different *Fusarium* species; Table S1: Pre-tests: The root exudates of *A. fistulosum* L. were used to perform inhibition tests on all isolated *Fusarium*; Table S2: Sequencing results of the three DNA sequences of *Fusarium solani* isolate HBH 08 and the corresponding sequence numbers.
