# Supplementary material for: Allium fistulosum L. Alleviates Apple Replant Disease by Suppressing Fusarium solani
Source: J Fungi (Basel). 2022 Oct 12;8(10):1071. doi: 10.3390/jof8101071 (PMC9605486; doi:10.3390/jof8101071)
Supplement: Supplementary file 1 [file jof-08-01071-s001.zip › jof-1937632-supplementary.pdf]

Table S1. Pre-tests: The root exudates of *A. fistulosum* L. were used to perform inhibition tests on all isolated *Fusarium*.

Treatment	Mycelial growth inhibition rate							Spore germination inhibition rate						
	F. equiseti	*F. lacertarum*	*F. proliferatum*	*F. oxysporum*	*F. solani*	*F. sulawense*	*F. verticillioides*	*F. equiseti*	*F. lacertarum*	*F. proliferatum*	*F. oxysporum*	*F. solani*	*F. sulawense*	*F. verticillioides*
Root exudates of *Allium fistulosum L.*	42.61%	46.39%	31.15%	49.95%	47.53%	38.65%	45.17%	43.91%	45.90%	49.48%	52.58%	61.62%	37.32%	48.83%

Table S2. Sequencing results of the three DNA sequences of *Fusarium solani* isolate HBH 08 and the corresponding sequence numbers.

Locus	Sequence number	Sequence (5′-3′)
ITS	OP268218	GCCTGCGGAGGGATCATTACCGAGTTATACAACTCATCAACCCTGTGAACATACCTAAAACGTTGCTTCGGCGGGAACAG ACGGCCCTGTAACAACGGGCCGCCCCCGCCAGAGGACCCCTAACTCTGTTTTTATAATGTTTTTCTGAGTAAACAAGCAAA TAAATTAAAACTTTCAACAACGGATCTCTTGGCTCTGGCATCGATGAAGAACGCAGCGAAATGCGATAAGTAATGTGAATT GCAGAATTCAGTGAATCATCGAATCTTTGAACGCACATTGCGCCCGCCAGTATTCTGGCGGGCATGCCTGTTCGAGCGTCA TTACAACCCTCAGGCCCCCGGGCCTGGCGTTGGGGATCGGCGGAAGCCCCCTGTGGGCACACGCCGTCCCTCAAATACAGT GGCGGTCCCGCCGCAGCTTCCATTGCGTAGTAGCTAACACCTCGCAACTGGAGAGCGGCGCGGCCATGCCGTAAAACACC
TEF-1α	OP373202	CAACTTCTGAATGTTGACCTCGAATCAGGTAGGAATACCCGCTGAACTTAAGCATATCAAAA CTGCTCTGGCAAGTCGACCACCGTAAGTCAAACCCTCATCGCGATCTGCTTATCTCGGGTCGTGG AACCCCGCCTGGCATCTCGGGCGGGGTATTCATCATTCACTTCATGCTGACAATCATCTACAGAC CGGTCACTTGATCTACCAGTGCGGTGGTATCGACAAGCGAACCATCGAGAAGTTCGAGAAGGTT GGTGACATCTGCCCCCGATCGCGCCTTGATATTCCACATCGAATTCCCCGTCGAATTCCCTCCAT CGCGATACGCTCTGCGCCCGCTTCTCCCGAGTCCCAAAATTTTTGCGGTCCGACCGTAATTTTTTT GGTGGGGCATTTACCCCGCCACTCGGGCGACGTTGGACAAAGCCCTGATCCCTGCACACAAAAA CACCAAACCCTCTTGGCGCGCATCATCACGTGGTTCACGACAGACGCTAACCGGTCCAACAATA GGAAGCCGCTGAGCTCGGTAAGGGTTCCTTCAAGTACGCCTGGGTCCTTGACAAGCTCAAGGCC GAGCGTGAGCGTGGTATCACCATCGACATTGCCCTCTGGAAGTTCGAGACTCCCCGCTACTATGT CACCGTCATTGGTATGTTGCTGTCACCTCTCTCACACATGTCTCACCGCTAACATAAAAAAAAAC GCCAAA
RPB2	OP373203	TCTCTCGGTGTTGACAGATACACCTTTGCATCCACTCTTTCGCATTTGCGACGAACCAACACCCCTATTGGACGAGATGGAA AGCTCGCCAAGCCTCGTCAGCTACACAACACCCATTGGGGTCTGGTCTGTCCAGCCGAGACGCCTGAGGGTCAGGCTTGTG GTCTGGTCAAGAACTTGTCCCTGATGTGCTACGTCAGTGTCGGCTCTCCCTCCGAACCTCTGATTGAGTTCATGATCAACCG AGGTATGGAAGTCGTAGAAGAGTACGAGCCCCTGAGATACCCCCATGCTACCAAGATCTTTGTCAACGGTGTCTGGTGCGG TGTTCATTCAGACCCCAAGCATCTCGTCAGCCAAGTCCTGGACACACGACGAAAGTCGTACCTGCAGTATGAGGTGTCGCT TGTTCGTGACATTCGAGATCGAGAGTTCAAGGTCTTCTCCGACGCTGGCCGAGTCATGAGGCCGGTCTTCACGGTTCAGCA GGAGGATGACCACGAGTCTGGTATTGCCAAGGGAGCTTTGGTTCTGACCAAGGACCTTGTCAACAAGCTTGCTAAGGAGC AGGCGGAGCCACCAGAGGACCCATCAATGAAGATTGGATGGGAGGGTCTGATCCGAGCCGGAACCATCGAGTACCTCGA TGCCGAGGAAGAGGAGACGGCTATGATTTGCATGACTCCTGAGGATCTTGATCTCTATCGCATGCAAAAGGCTGGTTACGT

CGTAGATGACGATAACACGGACGACCCCAACAGGAGATTGAAGACCAAGACGAACCCCACAACTCACATGTACACTCAT
TGTGAGATTCACCCTAGTATGATTCTTGGCATTTGTGCCAGTATCATTCCCTTCCCCGATCACAACCAGGTATGTGCCCATGA
TTCAACGTGATGCCAGCGAAACTAACAATATGTAGTCAC

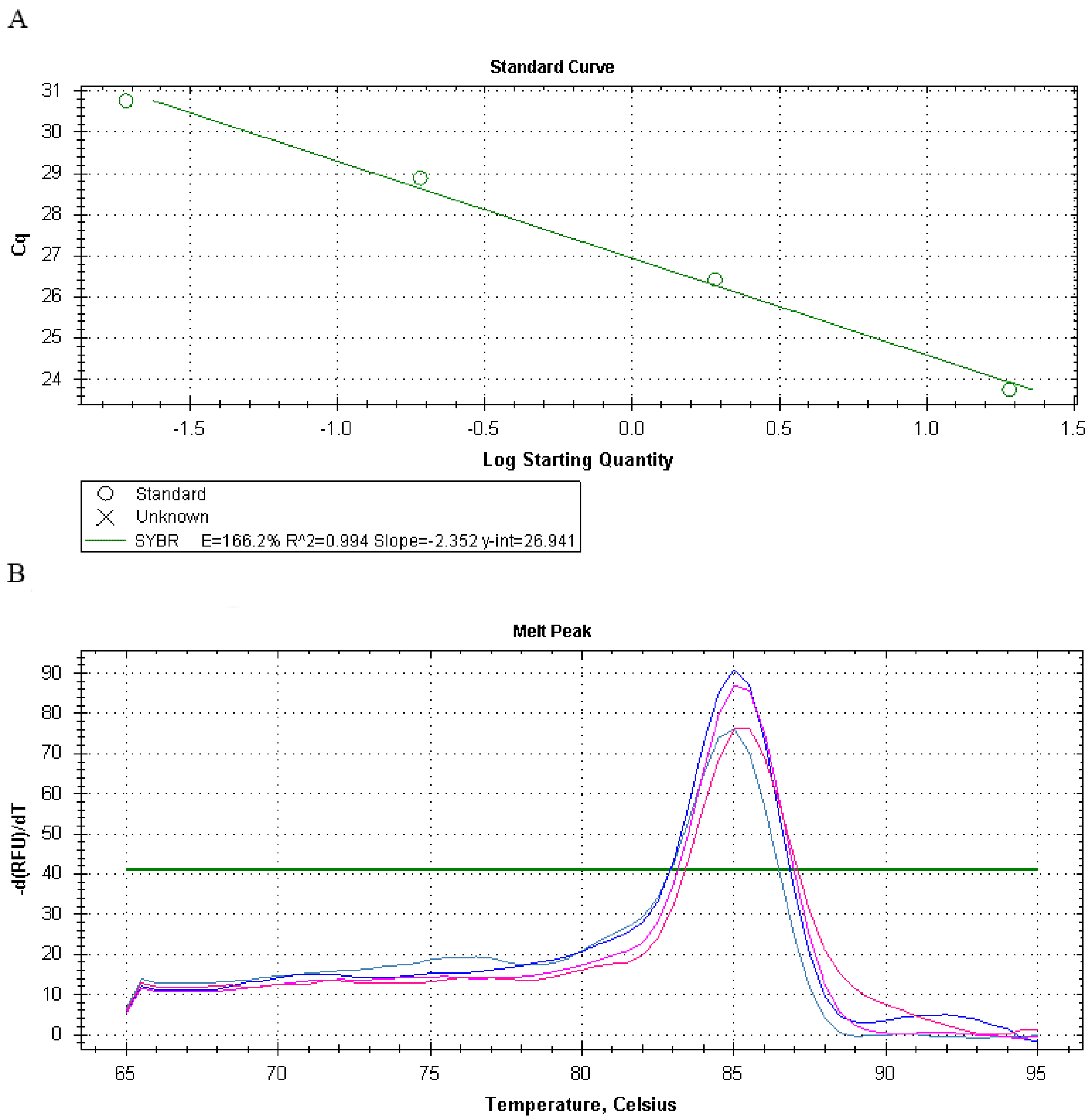

Figure S1. A: The standard curve for *Fusarium solani* isolate HBH 08 qRT-PCR; B: The melting curve for *Fusarium solani* isolate HBH 08 qPCR product.

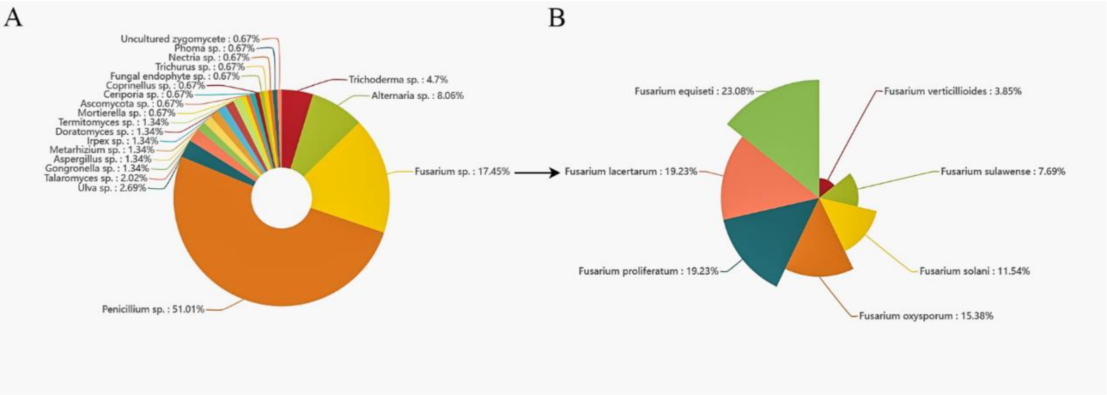

Figure S2. A: Relative abundance of 21 fungal genera isolated from diseased roots of apple trees; B: Percentage of isolation of different *Fusarium* species.
